# Thrombocytosis as a paraneoplastic syndrome in metastatic malignant peritoneal mesothelioma of biphasic morphology mimicking ovarian adenocarcinoma: A case report

**DOI:** 10.1002/ccr3.6974

**Published:** 2023-03-02

**Authors:** Moustafa S. Alhamadh, Rakan B. Alanazi, Osama Mohaamad Wadaan, Abdulrahman Yousef Alhabeeb, Mohammad Alkaiyat, Ohoud Zaid Aljarbou, Fouad Sabatin

**Affiliations:** ^1^ College of Medicine King Saud bin Abdulaziz University for Health Sciences, Ministry of the National Guard‐Health Affairs Riyadh Saudi Arabia; ^2^ King Abdullah International Medical Research Center Ministry of the National Guard‐Health Affairs Riyadh Saudi Arabia; ^3^ Department of Medical Oncology, King Abdulaziz Medical City Ministry of the National Guard‐Health Affairs Riyadh Saudi Arabia; ^4^ Department of Pathology, King Abdulaziz Medical City Ministry of the National Guard‐Health Affairs Riyadh Saudi Arabia

**Keywords:** malignant mesothelioma, medical oncology, metachronous malignancy, multiple primary malignancies, peritoneal mesothelioma

## Abstract

Malignant peritoneal mesothelioma (MPM) is a rare malignancy, presenting with non‐specific and potentially‐misleading manifestations. It represents a diagnostic pitfall as it mimics ovarian carcinoma. Maintaining a low diagnostic threshold, obtaining a detailed history, and utilizing immunohistochemical markers to diagnose MPM is crucial as early diagnosis and treatment might improve survival.

## Abstract 2

We report a case of multiple primary malignancies involving malignant peritoneal mesothelioma in a 55‐year‐old female with a remote history of radiotherapy for thyroid papillary adenocarcinoma. Her advanced disease made her unfit for chemotherapy and debulking surgery. She died of cardiac arrest 2 days after palliative care referral.

## 1 INTRODUCTION

Mesothelioma is an exceedingly rare and lethal malignancy of serosal membranes, with the visceral pleura being the most commonly affected followed by the peritoneum.^1^, ^2^ Malignant peritoneal mesothelioma accounts for roughly 10% of all mesotheliomas and has a 5‐year survival of 28% despite appropriate therapy.^3^, ^4^ It has a poor prognosis, especially in elderly male patients and patients with biphasic or sarcomatoid histopathologic morphology.^4^, ^5^ Several predisposing factors for malignant mesothelioma have been described in the literature, including mutations, such as BAP‐1, NF2, SETD2, TP53, DDX3X, ULK2, RYR2, CPAF45, SETDB1, and DDX51, and environmental exposures, such as asbestos, silicate fiber erionite, fluoredenite, balangeroite, carbon nanotubes, thorotrast dye, rock wool, slag wool, glass fiber, radiation, chronic inflammation, Papovavirus, and Simian virus.^6^, ^7^ Its clinical presentation is highly nonspecific and might present as abdominal mass, pain, distention, anorexia, early satiety, and weight loss eventually leading to bowel obstruction and cachexia.^8^ Cytoreductive surgery and hyperthermic intraperitoneal chemotherapy remain the mainstay of therapy for malignant peritoneal mesothelioma, but unfortunately, around 60% of the patients are noncandidate for surgery at the time of diagnosis.^4^ In this article, we report a case of multiple primary malignancies involving fatal metachronous malignant peritoneal mesothelioma and thyroid papillary adenocarcinoma in a 55‐year‐old female with a previous history of radiation therapy.

## 2 CASE PRESENTATION

A 55‐year‐old Saudi female gravida 10 para 9 + 1, known case of type 2 diabetes mellitus, hypertension, dyslipidemia, chronic endometritis, simple ovarian cyst, and thyroid papillary adenocarcinoma status post total thyroidectomy and radiotherapy, presented to our emergency department complaining of a localized right upper quadrant dull abdominal pain for 5 months accompanied by fatigue, night sweat, anorexia, and a month‐long history of nausea and vomiting. She reported an unintentional weight loss of 5 kg within the last 3 months. She denied a history of sick contact, fever, headache, chest pain, palpitations, syncope, joint pain, rectal bleeding, or urinary and bowel changes. Upon admission, she was on Metformin and Insulin Glargine for diabetes mellitus, Amlodipine for hypertension, Atorvastatin for dyslipidemia, Esomeprazole for occasional reflux, and Thyroxine due to her previous history of total thyroidectomy. Her past surgical history was notable for hysteroscopic polypectomy for menorrhagia 2 years ago and total thyroidectomy for papillary adenocarcinoma 15 years ago. Her family history was noncontributory. She is a housewife, and she denied any prolonged exposure to environmental toxins and carcinogens such as cigarette smoking, industrial smoke, and asbestos. On examination, she was ill‐appearing, distressed, and in pain. She was vitally unstable with a blood pressure of 97/83 mmHg and a heart rate of 116 bpm but a normal respiratory rate (20) and body temperature (36.4°C). She was alert, attentive, and oriented to time, place, and person. Her cardiopulmonary examination was unremarkable. Her abdomen was severely‐distended with a positive shifting dullness and fluid thrill, diffusely tender to palpation with a constant pain in the right upper quadrant, audible bowel sounds, and no distended veins or evidence of organomegaly. Both of her lower limbs were diffusely edematous without tenderness. She was admitted under internal medicine for diagnosis and treatment of ascites, and a drainage catheter was placed by the interventional radiology team.

Laboratory investigations were remarkable for microcytic hypochromic anemia (Hgb: 92 g/L, MCV: 74.7 fL, MCHC: 273 g/L), thrombocytosis (Platelets: 794 × 10^9/L), leukocytosis with a neutrophilic predominance (WBC: 13.8 × 10^9/L, Neutrophils: 12.1 × 10^9/L), mild hyperkalemia (5.3 mmol/L), hyperphosphatemia (1.63 mmol/L), hyponatremia (130 mmol/L), hypoalbuminemia (22 g/L), slightly elevated alkaline phosphatase (189 U/L), and gamma‐glutamyl transferase (41 U/L) without transaminitis (ALT: 24 U/L, AST: 27 U/L), and elevated lactate dehydrogenase (359 U/L), D‐dimer (2.65 mg/L), and inflammatory markers (ESR: 121 mm/h, CRP: 282 mg/L). Coagulation studies were within the normal range. Given her previous history of chronic endometritis, ovarian cyst, and papillary adenocarcinoma of the thyroid and her suspicious presentation of prolonged abdominal pain, unexplained weight loss, anorexia, fatigue, and night sweat, a metastatic gynecologic malignancy was suspected and tumor markers were ordered. Beta‐hCG (30 IU/L), CA 125 (663 U/mL), and CA 15‐3 (229 U/mL) were markedly elevated whereas alpha fetoprotein (<2.0 ng/mL), CEA (2 ng/mL), and CA 19.9 (4 U/mL) were within normal limits. Blood, urine, and ascitic fluid cultures yielded no microbial growth. Her serum‐ascites albumin gradient was 0.5 g/dL, probably reflecting peritoneal carcinomatosis. Ascitic fluid cytologic analysis showed reactive mesothelial cells, histocytes, and lymphocytes intermixed with few highly atypical cells worrisome for adenocarcinoma of ovarian origin.

**FIGURE 1:**
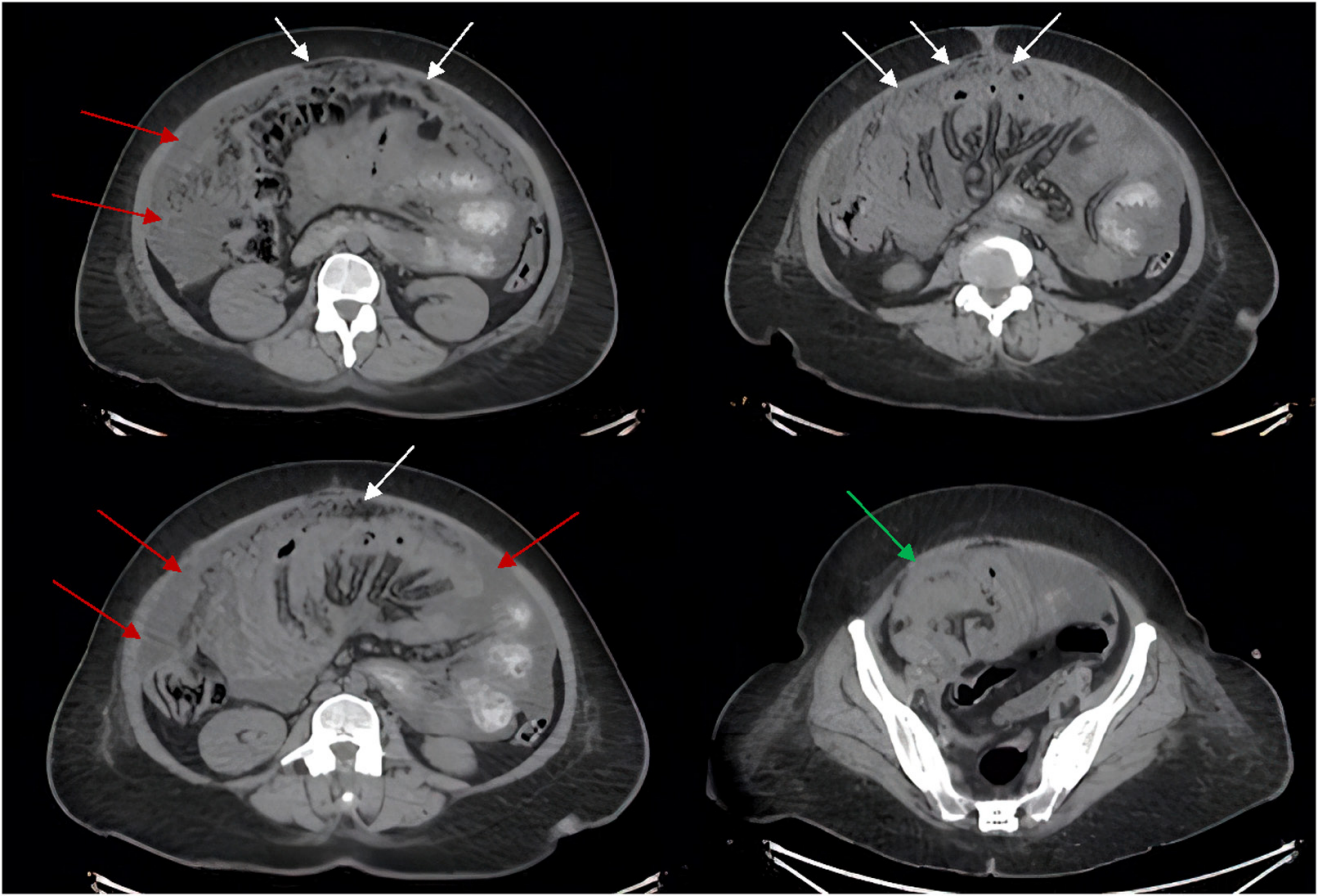
Cross‐sectional (transverse) abdominal and pelvic CT with contrast. It shows diffuse omental caking (White arrows) with thick enhancing peritoneal reflections (Red arrows) and soft tissue thickening of the right adnexa with a poorly defined ovary (Green arrow).

**FIGURE 2:**
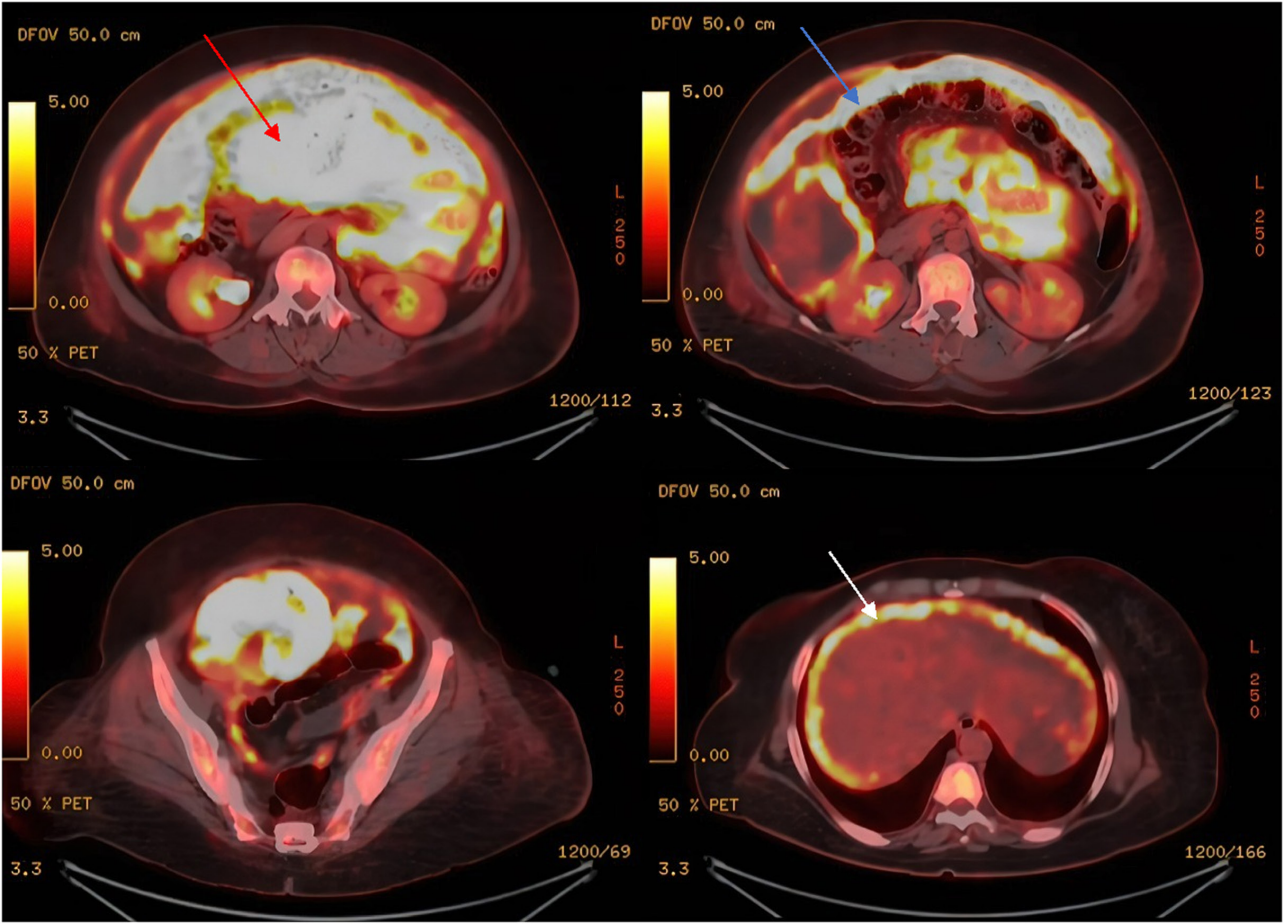
Cross‐sectional (transverse) abdominal/pelvis PET/CT with FDG. It shows an intensely increased FDG uptake around the peritoneal lining (White arrow) with peritoneal thickening (Blue arrow) and omental caking (Red arrow).

**FIGURE 3:**
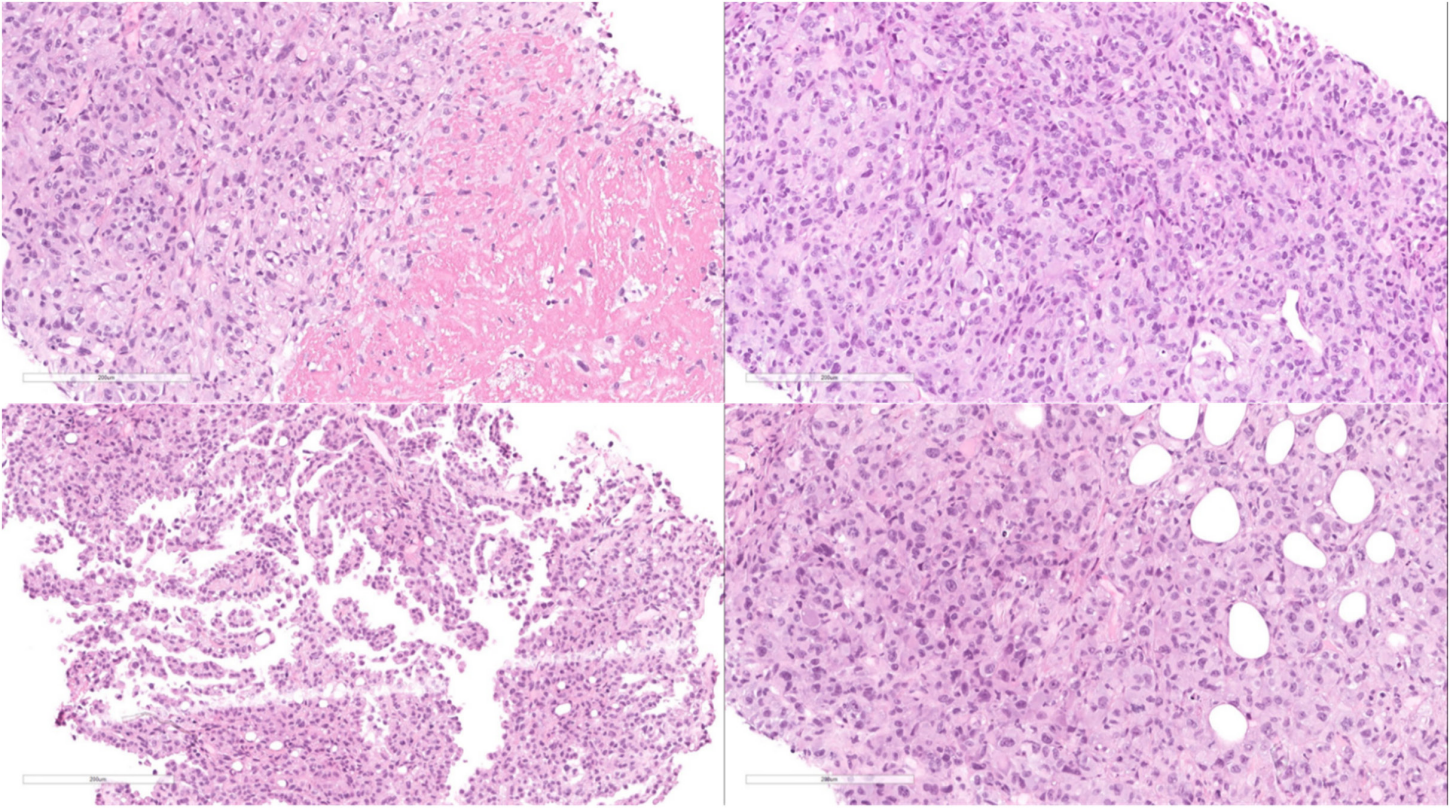
Histopathology of the H&E‐stained sample (magnification ×200) shows papillary pattern lined by relatively bland cells with eosinophilic cytoplasm and a weak solid growth pattern of poorly differentiated pleomorphic cells.

**FIGURE 4:**
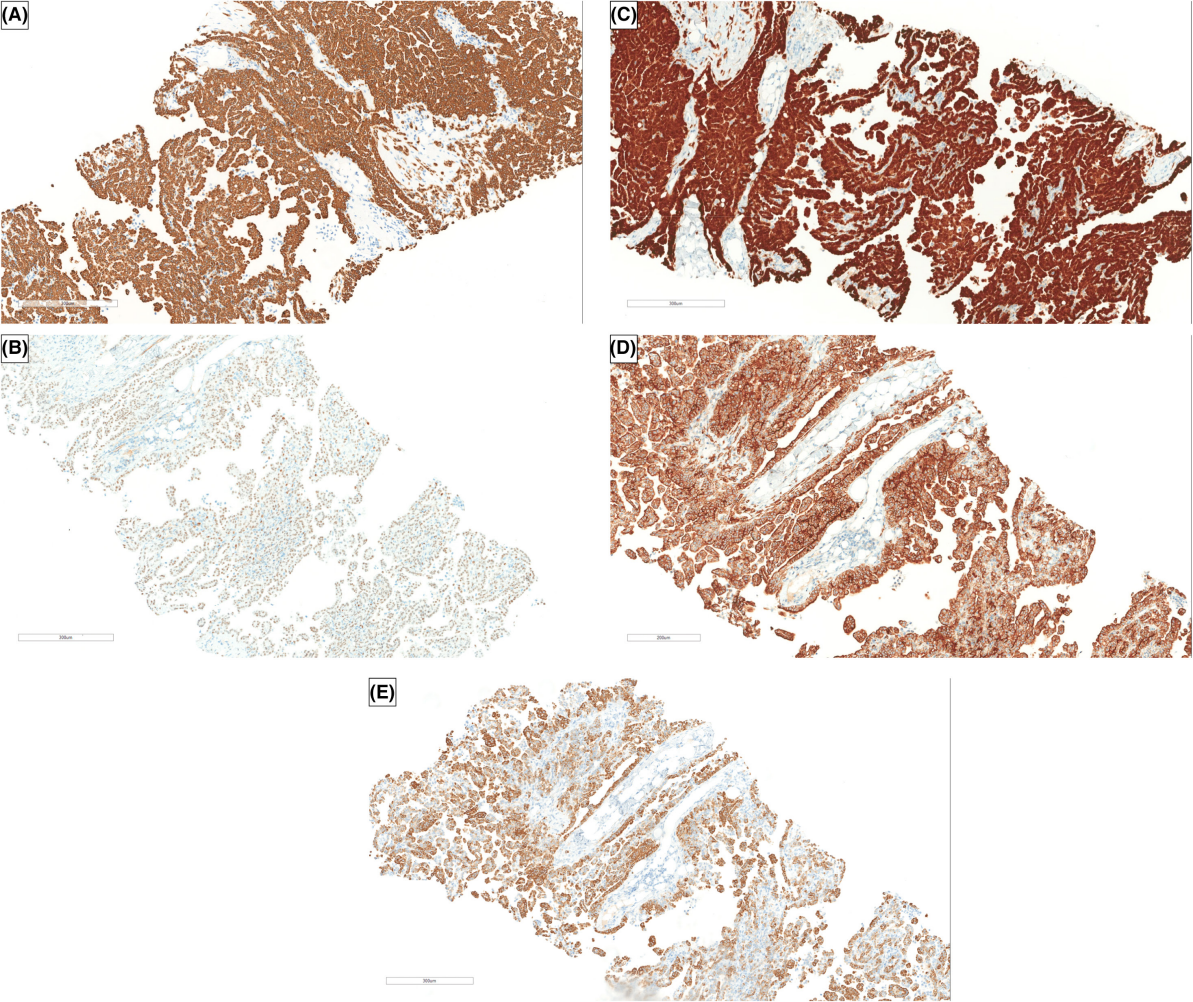
Histopathological tissues are positive for PAN CK (A), WT1 (B), Calretinin (C), D2‐40 (D), and CK5/6 (E) immunohistochemical markers.

**FIGURE 5:**
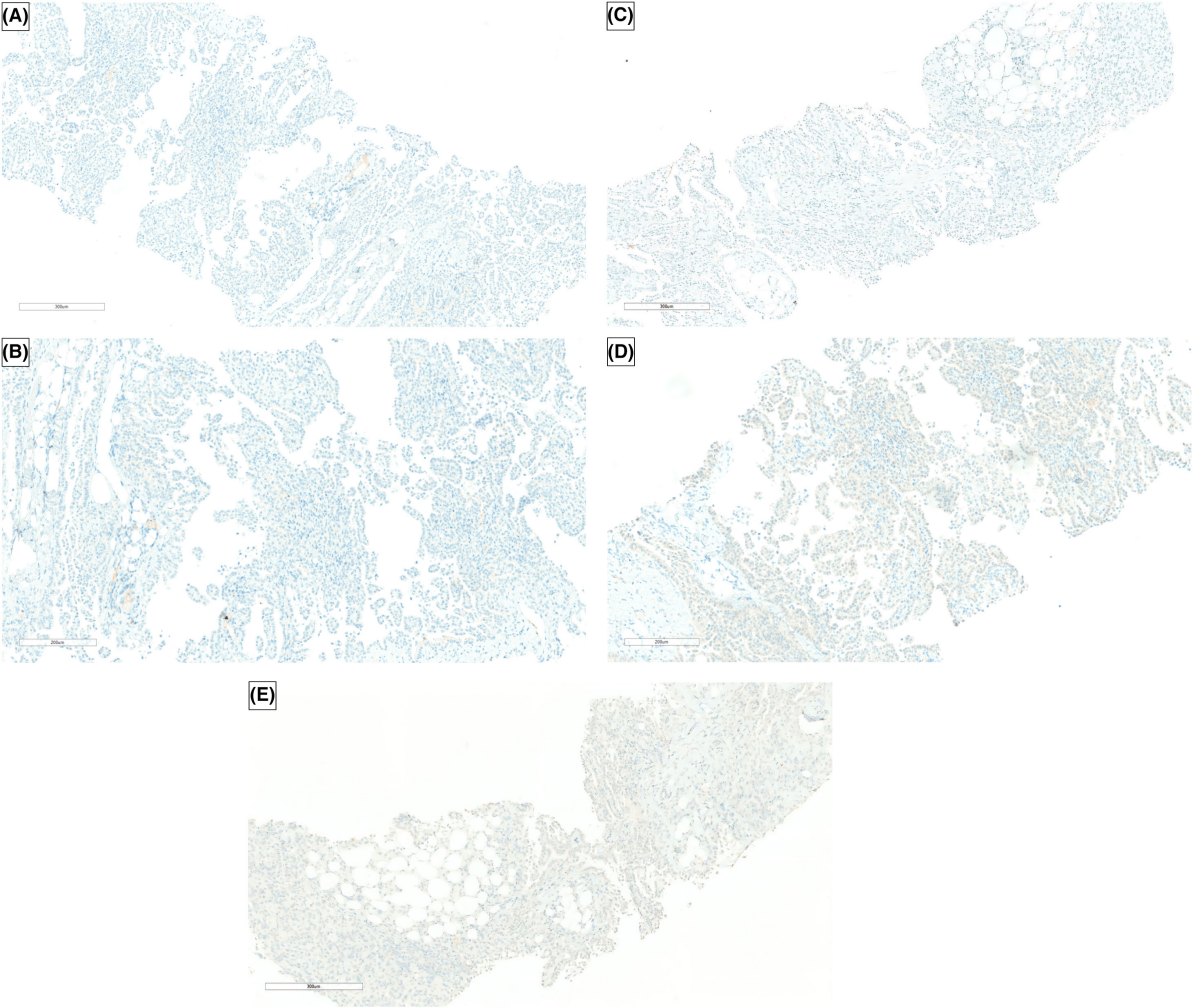
Histopathological tissues were negative for the following adenocarcinoma markers: MOC‐31 (A), BerEP‐4 (B), ER (C), Thyroglobulin (D), and TTF‐1 (E).

Supine and upright abdominal radiographs were unremarkable. Abdominal CT with contrast showed diffuse omental caking with thick enhancing peritoneal reflections associated with moderate ascites and mesenteric root fluid collection. Also, there were enlarged retrocaval, paraaortic, and internal mammary lymph nodes. In addition, soft tissue thickening of the right adnexa with a poorly defined ovary was noted, which is also suspicious of ovarian malignancy (Figure 1). PET/CT scan with FDG demonstrated an intensely hypermetabolic peritoneal and omental thickening, para‐caval lymph node, liver foci, and right adnexa, suggestive of metastatic disease (Figure 2). The gynecologic oncology team was counseled regarding these findings, and after ruling out ovarian neoplasms, a tru‐cut needle biopsy of the omental caking was done. Histopathological evaluation demonstrated a dominant papillary pattern lined by relatively bland cells with eosinophilic cytoplasm and a weak solid pattern of poorly differentiated pleomorphic cells (Figure 3). These cells were positive for PAN CK, WT1, Calretinin, D2‐40, and CK5/6 (Figures 4A–E) but negative for adenocarcinoma markers such as MOC‐31, Ber‐EP4, ER, thyroglobulin, and TTF‐1 (Figures 5A–E).

Based on the imaging, histopathological, and immunohistochemical findings, a diagnosis of metastatic malignant peritoneal mesothelioma of biphasic morphology was made. Due to her advanced metastatic disease and overall poor general health, medical and surgical oncology referred her to palliative care as she was unfit for chemotherapy and debulking surgery. She received a high dose of morphine and lorazepam without a significant pain improvement, and 2 days later, she died of cardiac arrest.

## 3 DISCUSSION

Multiple primary malignancies are defined as the occurrence of two or more independent malignancies in the same or different organs. They are classified as synchronous or metachronous based on the time interval between malignancy diagnoses. A metachronous malignancy is a second malignancy occurring at least 6 months after the first malignancy.^9^ In this article, we report a rare case of metachronous diffuse malignant peritoneal mesothelioma mimicking ovarian adenocarcinoma with peritoneal metastasis.

We believe that this case is interesting due to the following reasons: (1) Malignant peritoneal mesothelioma is a very rare malignancy with a nonspecific clinical presentation. Because of that, this case highlights the importance of keeping it within the differential diagnosis of malignant ascites, especially in patients with previous exposure to radiation as it is a well‐established risk factor for malignant mesothelioma.^6^ Radiation is a known pancarcinogen, and abdominal mesothelioma has been reported in patients treated with radiation for mainly Hodgkin and non‐Hodgkin lymphomas, Wilms tumor, and breast cancer, but not thyroid papillary adenocarcinoma.^10^, ^11^, ^12^ It is worth‐mentioning that the possibility of these two malignancies being related is almost negligible as the patient was exposed to radiation in her neck, which is away from the peritoneal cavity. Regardless, this case still meets the definition of multiple primary malignancies. (2) To the best of our knowledge, malignant peritoneal mesothelioma has not been reported before as a metachronous malignancy to thyroid papillary adenocarcinoma. (3) Malignant peritoneal mesothelioma must be distinguished from ovarian serous and peritoneal serous carcinomas to avoid unnecessary workup and expedite the diagnostic process.^13^ In addition, the treatment options and prognosis of these malignancies are different. (4) Roughly, 30 cases of malignant peritoneal mesothelioma mimicking ovarian cancer have been reported in the literature, making this clinical presentation rare and of a high educational value.^13^ (5) Unlike epithelioid malignant mesothelioma, biphasic, as in our case, deciduoid, and sarcomatoid morphologies are exceedingly rare. In fact, biphasic malignant mesothelioma is probably the rarest subtype. Based on a recent review of 76 patients with malignant mesothelioma, epithelioid morphology was the commonest subtype, followed by sarcomatoid, deciduoid, and biphasic morphologies, accounting for 74.3%, 12.8%, 8.6%, and 4.3%, respectively.^14^ To the best of our knowledge, only eight cases of malignant abdominal mesothelioma of biphasic morphology have been reported in the literature, all of which were localized.^15^ Our patient had a metastatic disease that diffusely involved the peritoneum, several lymph nodes, and liver. (6) This might be the first reported case of malignant peritoneal mesothelioma in the Kingdom of Saudi Arabia.

This case is limited by the absence of genetic testing and the weak association between neck radiation and development of malignant mesothelioma. BRCA1‐associated protein 1 (BAP1) is a tumor suppressor gene and its loss has been reported in malignant mesothelioma.^16^ Recently, a novel germline BAP1 mutation (c.1777C > T) has been described in a family whose members were affected by early‐onset melanocytic neoplasms but also developed other types of cancer including thyroid papillary cancer.^17^

Mesothelioma is an insidious lethal malignancy arising from mesothelial surfaces, with the pleura being the most commonly afflicted (65%–70%), followed by the peritoneum (10%–30%), tunica vaginalis, and pericardium (1%–2%).^3^ Malignant abdominal mesothelioma represents a diagnostic dilemma as it presents with non‐specific manifestations.^8^ Our patient had a long history of generalized abdominal pain that became localized to the right upper quadrant later on, probably due to liver involvement. She also reported unexplained weight loss and severe loss of appetite. The literature identifies weight loss and abdominal pain, and distention as the most common complaints, accounting for 69%, 35%, and 31%, respectively.^18^ Only a small portion of the patients may develop night sweats, as in our case, and fever. Ascites is reported in up to 77% of the cases.^18^ In addition, associated paraneoplastic syndromes, such as thrombocytosis, hypercoagulability, neuropathy, hypoglycemia, and cachexia, have been described in patients with malignant mesothelioma.^3^, ^19^ Our patient had severe thrombocytosis. Thrombocytosis is a surrogate for tumor aggressiveness and has been associated with worse survival despite appropriate therapy.^20^

Although malignant peritoneal mesothelioma and ovarian carcinoma are barely distinguishable morphologically and share many diagnostic markers such as keratins and vimentin, there are some biological and immunohistochemical differences to distinguish both cancers. To begin with, compared to ovarian carcinoma, malignant peritoneal mesothelioma has a higher expression of the nerve growth factor receptors p75 and p‐TrKA and the angiogenic molecules bFGF but lower expression of the laminin receptor 67‐kDa and α6 integrin, the angiogenic molecules VEGF and IL‐8, and the human leukocyte antigen G.^21^ Moreover, the role of calretinin and thrombomodulin as positive markers and Ber‐EP4, MOC‐31, CA19‐9, and estrogen receptor as negative markers in differentiating malignant abdominal mesothelioma and ovarian carcinoma has been established in multiple studies.^22^, ^23^ These markers have a relatively high sensitivity and specificity for diagnosing malignant peritoneal mesothelioma, especially in the presence of a history that increases the risk of this malignancy such as radiation or asbestos exposure.

In our case, the diagnosis of malignant peritoneal mesothelioma was confirmed by histopathological examination of a core needle biopsy of the omental cacking. The cells came positive for PAN CK, WT1, Calretinin, D2‐40, Podoplanin, and CK5/6. These markers are not 100% specific but have high sensitivities. To emphasize, the most sensitive marker is Calretinin (100%), followed by WT1 (94%), CK5/6 (89%), and D2‐40 (80%).^24^

## 4 CONCLUSION

Malignant peritoneal mesothelioma is an extremely rare but rapidly progressive and highly fatal malignancy. It rarely presents with paraneoplastic syndromes, and it has non‐specific manifestations, which makes it a diagnostic dilemma. Clinicians should maintain a low threshold of its diagnosis, especially among patients with a previous history of radiation or asbestos exposure. Knowledge about malignant peritoneal mesothelioma signs and symptoms, risk factors, and associated paraneoplastic syndromes is crucial as early detection and treatment might improve survival.

## AUTHORS CONTRIBUTIONS

**MSA** (MBBS) was responsible for conceiving the report idea and acquiring the patient's data. **MSA**, **RBA**, **OMW**, and **AYA** (MBBS) were responsible for writing as well as reviewing the original manuscript. **MA** (CCRP, CCRC) and **OZA** (MD) were responsible for providing the required images/figures. **MA** (CCRP, CCRC), **FS**, and **OZA** (MD) critically reviewed the final form of the manuscript and supervised the whole process. **All authors** contributed to literature search and approved the final form of this paper, and are responsible for its content.

## FUNDING INFORMATION

No funding was needed for this paper.

## CONFLICT OF INTEREST STATEMENT

The authors declare no conflict of interest.

## ETHICS STATEMENT

This case report does not involve any studies with human subjects or animals, and therefore, it does not need ethical approval. The confidentiality of the patient was protected and no names nor any identifiers were mentioned.

## CONSENT

A written informed consent was obtained from the patient's son for the purpose of publication as she passed away. The importance of reporting her case for educational purposes was explained, and the patient's son agreed.

## Data Availability

The data that support the findings of this study are available on request from the corresponding author. The data are not publicly available due to privacy or ethical restrictions.
